# Relative deficiency in interferon‐γ‐secreting CD4+ T cells is strongly associated with poorer COVID‐19 vaccination responses in older adults

**DOI:** 10.1111/acel.14099

**Published:** 2024-02-05

**Authors:** Vanda W. T. Ho, Low Heng Boon, Jianzhou Cui, Zhou Juequn, Bhuvaneshwari Shunmuganathan, Rashi Gupta, Nikki Y. J. Tan, Xinlei Qian, Kiren Purushotorman, SCOPE Cohort Study Group, Siti Naqiah Amrun, Yun‐Shan Goh, Matthew Zi‐Rui Tay, Angeline Rouers, Zi Wei Chang, Nicholas Kim‐Wah Yeo, Yi‐Hao Chan, Pei Xian Hor, Chiew Yee Loh, Yuling Yang, Anthony Torres Ruesta, Vanessa Neo, Wendy Yehui Chen, Estelle Yi‐Wei Goh, Alice Soh‐Meoy Ong, Adeline Chiew Yen Chua, Samantha Nguee, Yong Jie Tang, Weiyi Tang, Joel Xu En Wong, Anna Loo, Liang Hui Loo, Andrea Chua, Siew‐Wai Fong, Laurent Renia, Lisa F. P. Ng, Veronique Angeli, Jinmiao Chen, Brian K. Kennedy, Catherine W. M. Ong, Paul A. Macary

**Affiliations:** ^1^ Division of Geriatric Medicine, Department of Medicine National University Hospital Singapore Singapore; ^2^ Immunology Translational Research Program, Yong Loo Lin School of Medicine National University of Singapore Singapore Singapore; ^3^ Infectious Diseases Translational Research Program, Yong Loo Lin School of Medicine National University of Singapore Singapore Singapore; ^4^ Department of Microbiology and Immunology, Yong Loo Lin School of Medicine National University of Singapore Singapore Singapore; ^5^ Department of Medicine, Yong Loo Lin School of Medicine National University of Singapore Singapore Singapore; ^6^ NUS Immunology Program, Life Sciences Institute National University of Singapore Singapore Singapore; ^7^ NUS‐Cambridge Immune Phenotyping Centre (NCIPC), Life Sciences Institute National University of Singapore Singapore Singapore; ^8^ Metabolic Core, Yong Loo Lin School of Medicine National University of Singapore Singapore Singapore; ^9^ Antibody Engineering Programme, Life Sciences Institute National University of Singapore Singapore Singapore; ^10^ A*STAR Infectious Diseases Labs (A*STAR ID Labs) Agency for Science, Technology and Research (A*STAR) Singapore Singapore; ^11^ Lee Kong Chian School of Medicine Nanyang Technological University Singapore Singapore; ^12^ Singapore Immunology Network (SIgN) Agency for Science, Technology and Research (A*STAR) Singapore Singapore; ^13^ Healthy Longevity Translational Research Programme, Yong Loo Lin School of Medicine National University of Singapore Singapore Singapore; ^14^ Department of Biochemistry and Physiology, Yong Loo Lin School of Medicine National University of Singapore Singapore Singapore; ^15^ Institute for Health Innovation and Technology National University of Singapore Singapore Singapore; ^16^ Division of Infectious Diseases, Department of Medicine National University Hospital Singapore Singapore

**Keywords:** antigen‐specific T‐cell response, COVID‐19 mRNA vaccine, IFN‐γ, immunosenescence, older adults

## Abstract

Although the two‐dose mRNA vaccination regime provides protection against SARS‐CoV‐2, older adults have been shown to exhibit poorer vaccination responses. In addition, the role of vaccine‐induced T‐cell responses is not well characterised. We aim to assess the impact of age on immune responses after two doses of the BNT162b2 mRNA vaccine, focussing on antigen‐specific T‐cells. A prospective 3‐month study was conducted on 15 young (median age 31 years, interquartile range (IQR) 25–35 years) and 14 older adults (median age 72 years, IQR 70–73 years). We assessed functional, neutralising antibody responses against SARS‐CoV‐2 variants using ACE‐2 inhibition assays, and changes in B and T‐cell subsets by high‐dimensional flow cytometry. Antigen‐specific T‐cell responses were also quantified by intracellular cytokine staining and flow cytometry. Older adults had attenuated T‐helper (Th) response to vaccination, which was associated with weaker antibody responses and decreased SARS‐CoV‐2 neutralisation. Antigen‐specific interferon‐γ (IFNγ)‐secreting CD4+ T‐cells to wild‐type and Omicron antigens increased in young adults, which was strongly positively correlated with their neutralising antibody responses. Conversely, this relationship was negative in older adults. Hence, older adults' relative IFNγ‐secreting CD4+ T cell deficiency might explain their poorer COVID‐19 vaccination responses. Further exploration into the aetiology is needed and would be integral in developing novel vaccination strategies and improving infection outcomes in older adults.

## Graphical abstract

We conducted a prospective 3‐month immunophenotyping of 15 young (median 31 years) and 14 older adults (median 72 years) BNT162b2 vaccinees. Interferon‐γ(IFNγ)‐secreting CD4+ T‐cells to wild‐type and Omicron antigens increased in young adults, which was strongly positively correlated with their neutralising responses. Conversely, this relationship was negative in older adults. Hence, older adults' relative IFNγ‐secreting CD4+ T‐cell deficiency might explain their attenuated T‐helper response to vaccination, which in turn was associated with weaker neutralising antibody responses.

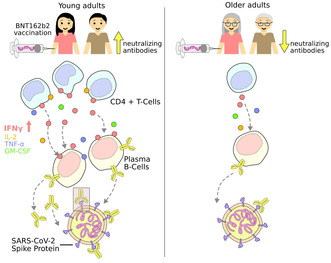

**Abbreviations**
APC: antigen presenting cells
EAE: experimental autoimmune encephalomyelitis
GM‐CSF: granulocyte‐macrophage colony‐stimulating factor
IFNγ: interferon‐gamma
Ig: immunoglobulin G
IL: interleukin
IQR: interquartile range
MBC: memory B cell
MHC: major histocompatibility complex
mRNA: messenger ribonucleic acid
NC: nucleocapsid
SD: standard deviation
Tfh: T‐follicular helper
Th: T‐helper
TNFα: tumour necrosis factor‐alpha
VOCs: variants of concern
WT: wild‐type

## 1 INTRODUCTION

Older adults, especially those who are frail, had notably worse outcomes in the COVID‐19 pandemic. Due to a waning vaccination response with age, this disparity continues to the present day when COVID‐19 vaccination has been applied globally. A major contributor to this attenuated vaccination response is immunosenescence, the age‐related physiological alterations of cellular and humoral immunity and inflammaging, a state of persistent elevation of circulating pro‐inflammatory proteins secondary to continuous antigenic stimulation (Fulop et al., 2017). Initial trials failed to include older adults (Helfand et al., 2020), and only recently has there been an interrogation of the older adults' immune system in response to the COVID‐19 vaccination. Collier and colleagues found reduced somatic hypermutation in class‐switched cells, and interferon‐γ (IFNγ) and interleukin (IL)‐2 production by SARS‐CoV‐2 spike‐specific CD4+ T‐cells (Collier et al., 2021). However, despite the importance of T‐cells in immunosenescence, their role in the age‐related differences in the COVID‐19 vaccine responses is not well characterised. Thus, we assessed the impact of age on neutralisation, humoral and cellular immune response after 2 doses of the BNT162b2 messenger ribonucleic acid (mRNA) vaccine in community‐dwelling adults, with a focus on the functionality of antigen‐specific T‐cells.

**FIGURE 1:**
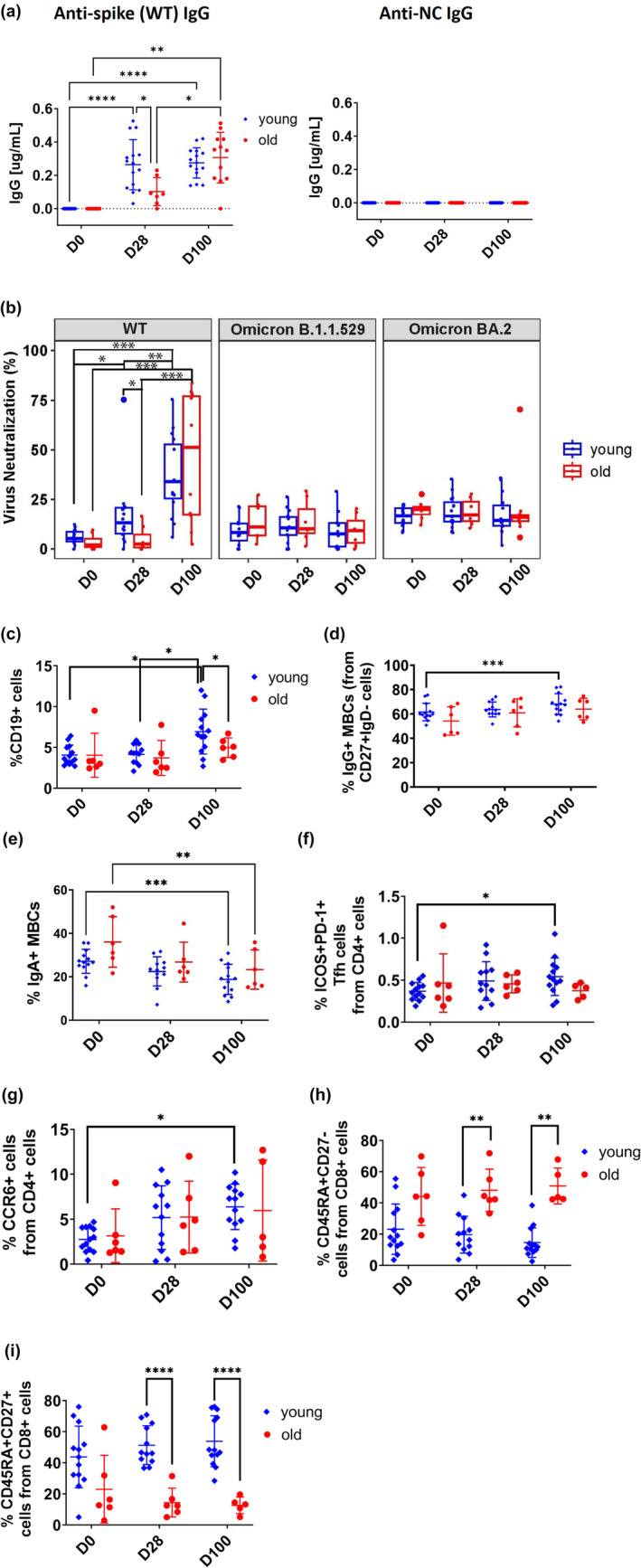
Immune responses to SARS‐CoV2 mRNA vaccination. For humoral responses: (a) Anti‐Spike IgG titres and nucleocapsid (NC) test of infection (b) Virus neutralisation assays against SARS‐CoV2 wild‐type, Omicron B.1.1.529 and Omicron BA.2 variants for young (*n* = 15) and old (*n* = 12) pre/post vaccinees. (c) Frequency of total B‐cells (CD19 + CD3‐CD14‐), (d) IgG+ (CD19+ CD27+ IgG+ IgM‐) and (e) IgA+ (CD19+ CD27+ IgA+ IgG‐ IgM‐) memory B‐cells in pre/post vaccinees. For cellular responses: (f) Frequency of ICOS+ PD‐1+ Tfh cells (ICOS+PD‐1 + CXCR5 + CD4+) (g) Th17 (CCR6 + CXCR3‐CCR4‐CD4+), (h) TEMRA (CD45RA + CD27‐CD8+) and (i) naive cells (CD45RA + CD27 + CD8+) in young (*n* = 15) and old (*n* = 7) pre/post vaccinees. Sidak's multiple comparison correction was used for comparing young versus old, Tukey's multiple comparison correction was used for comparing time‐points within the age groups. *p* values were determined by two‐way ANOVA with Sidak's and Tukey's multiple comparison corrections; *p < 0.05; **p < 0.01; ***p < 0.001. Bars represent mean ± standard deviation.

We recruited 22 COVID‐19 community‐dwelling adults, 15 young (median [interquartile range (IQR)] age, 31 years [25–35]) and 14 older (72 years [70–73]). They went on to receive two BNT162b2 vaccine doses (Table S1) and were followed up for 3 months. About half (51.7%) were male, 96.6% were Chinese. Older adults performed poorer on functional tests. All participants did not have detectable COVID‐19 infection throughout the course of this study. This was screened by the detection of antibodies to the nucleocapsid (NC) protein which helps to identify individuals with an adaptive immune response to SARS‐CoV‐2 (Figure 1a). Younger adults had significantly more anti‐spike immunoglobulin G (IgG) compared to older adults at day 28 (young, mean ± standard deviation (SD): 0.26 ± 0.15 vs. old: 0.10 ± 0.08, *p* = 0.01), which was seen previously (Collier et al., 2021; Goel et al., 2021; Sureshchandra et al., 2021).

## 2 DEVELOPMENT OF NEUTRALISING ANTIBODY RESPONSES TO SARS‐CoV‐2 VOCs AFTER TWO DOSES OF BNT162b2 mRNA VACCINE WAS IMPAIRED IN OLDER ADULTS

To assess the humoral immune response to COVID‐19 vaccination, blood was collected from SARS‐CoV‐2–naive volunteers prior to mRNA vaccination (D0), 1 month after the first vaccination (D28) and 2 months after the second vaccination (D100). Vaccination was able to induce neutralising antibody responses in young adults against the wild‐type (WT) strain as well as the Alpha, Beta, Gamma and Delta variants after the first vaccination, and was further significantly increased after the second vaccination (WT, mean ± SD: young at D0 6.14 ± 3.53 vs. D100 36.28 ± 19.83, *p* < 0.0001 compared to old at D0 3.48 ± 3.94 vs. D100 45.86 ± 31.70, *p* = 0.01; Alpha: young at D0 8.80 ± 4.29 vs. D100 35.87 ± 17.23, *p* < 0.0001 compared to old at D0 7.44 ± 2.88 vs. D100 25.42 ± 19.64; Beta: young at D0 6.68 ± 2.58 vs. D100 25.93 ± 12.21, *p* < 0.0001 compared to old at D0 8.05 ± 3.84 vs. D100 22.52 ± 15.46; Gamma: young at D0 7.83 ± 2.31 vs. D100 22.46 ± 9.67, *p* < 0.0001 compared to old at D0 7.41 ± 2.30 vs. D100 18.05 ± 11.65; Delta: young at D0 6.87 ± 2.80 vs. D100 41.45 ± 20.15, *p* < 0.0001 compared to old at D0 8.75 ± 2.60 vs. D100 30.88 ± 27.09) (Figure 1b and Figure S3, Table S2 and Figure S2). Although increases in neutralising antibody responses were also observed in the older group, these changes were not significant in all strains except WT. An increase in neutralising antibody responses against Omicron variants B.1.1529 or BA.2 was not observed in either group after vaccination.

In B‐cells, vaccination resulted in a significant expansion of CD19+ B cells in younger but not in older adults (young, mean ± SD at D0: 4.52 ± 1.93 vs. D100: 7.11 ± 2.53, *p* = 0.047) (Figure 1c, Table S3 and Figure S1). Similarly, younger adults had significantly increased IgG+ memory B‐cells (MBCs) at D100 compared to baseline (young, mean ± SD at D0 61.57 ± 7.22 vs. D100 68.07 ± 8.53, *p* = 0.0006), but this increase older adults was not significant across the time‐points (Figure 1d). In contrast, both young and older participants showed a reduction in IgA+ MBCs at D100 compared to D0 (young, mean ± SD at D0 27.12 ± 5.54 vs. D100 18.80 ± 6.92, *p* = 0.0002 compared to old at D0 36.03 ± 11.67 vs. D100 23.28 ± 9.07, *p* = 0.0055) (Figure 1e).

## 3 OLDER ADULTS HAD WEAKER CD4+ T‐HELPER RESPONSES TO VACCINATION AND MORE CD8+ T‐CELL SENESCENCE

Although the humoral response plays a major role in protection against initial infection, cellular immunity might help to limit disease progression when neutralising antibody titres decline. The effects of mRNA vaccination were thus examined in T‐cells.

Among the CD4+ T‐cells, only the young showed significant increases in the frequencies of circulating T‐follicular helper (TfH, ICOS+PD‐1 + CXCR5 + CD4+) cells (young, mean ± SD at D0 0.37 ± 0.11 vs. D100 0.54 ± 0.22, p = 0.03 compared to old at D0 0.47 ± 0.35 vs. D100 0.37 ± 0.09, Figure 1f, Table S3 and Figure S1) and CCR6+ T‐helper 17 (Th17, CCR6 + CD4+) cells with vaccination (young, mean ± SD at D0 2.76 ± 1.37 vs. D100 6.39 ± 2.53, *p* = 0.0001 compared to old at D0 3.16 ± 2.98 vs. D100 5.97 ± 5.62, Figure 1g). These CD4+ T‐cell differences may contribute to the disparate humoral responses seen.

For CD8+ cells, a higher frequency of CD45RA + CD27‐Effector Memory‐Expressing CD45RA (T_EMRA_) CD8+ T cells was observed in older adults compared to the young at D28 (young, mean ± SD at D28 19.62 ± 11.79 compared to old at D28 48.12 ± 13.52, *p* = 0.005) and D100 (young, mean ± SD at D100 14.58 ± 9.47 compared to old at D100 50.84 ± 11.55, *p* = 0.002, Figure 1h). Older adults also exhibited less CD45RA+ CD27+ naive CD8+ cells at D28 (young, mean ± SD at D28 51.29 ± 12.48 compared to old at D28 14.39 ± 9.23, *p* < 0.0001) and D100 (young, mean ± SD at D100 53.86 ± 16.34 compared to old at D100 12.62 ± 5.29, *p* < 0.0001, Figure 1i). However, frequencies of both CD45RA+ CD27+ naive and CD45RA + CD27‐ T_EMRA_ CD8+ cells remained unchanged in both groups after vaccination.

## 4 OLDER ADULTS HAD IMPAIRED IFNγ+ EXPRESSING CD4+ T‐HELPER CELLS TO WILD‐TYPE AND OMICRON BA.2 ANTIGENS

Given the well‐established impact of immunosenescence on T cells, we performed intracellular cytokine staining and flow cytometry to characterise antigen‐specific T‐cell effector responses after vaccination. CD4+ T‐cells that were responsive to antigen stimulation were defined as CD4 + CD154+ T‐cells. Older adults did not show a significant expansion of CD4+ CD154+ T‐cells in response to WT and Omicron BA.2 spike, and NC antigens over time. In contrast, a significant increase in the frequency of CD4 + CD154+ T‐cells was observed in young adults at D100 in response to WT (mean ± SD, D0 0.059 ± 0.069 vs. D100 0.21 ± 0.19, *p* = 0.050, Tables S3 and S4), Omicron (D0 0.052 ± 0.095 vs. D100 0.22 ± 0.21, *p* = 0.030) and NC (mean ± SD, D0 0.047 ± 0.073 vs. D100 0.16 ± 0.14, *p* = 0.034) antigen stimulations. The frequency of antigen‐responsive cells was increased in the young adults after the first dose (D28) but the increase was not significant.

**FIGURE 2:**
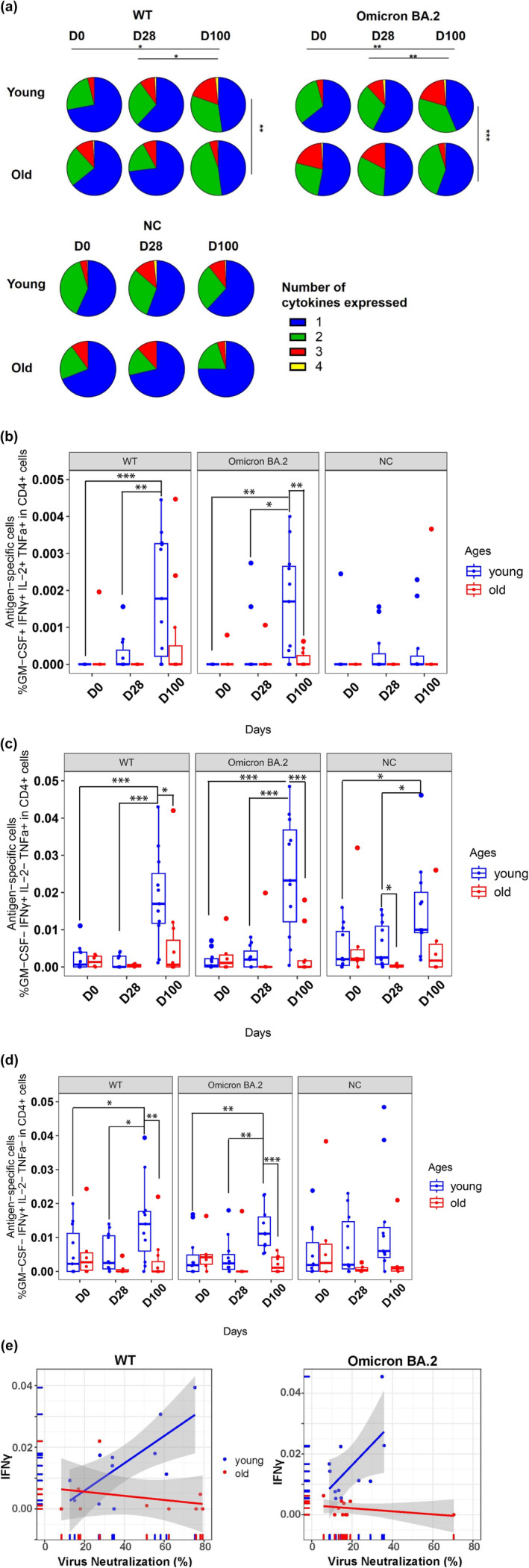
Antigen‐specific T‐cell responses. (a) CD4+ T cell cytokine expression after stimulation with WT, Omicron BA.2 and nucleocapsid antigens. Proportion of CD4+ T cells expressing 1, 2, 3 or 4 cytokines after stimulation with WT and Omicron BA.2 spike proteins, and nucleocapsid. *p*‐values were determined by Boolean analysis; *p < 0.05. Spike‐specific CD4+ T‐cell effector responses to SARS‐COV2 mRNA vaccination: frequencies of CD4+ T‐cells expressing (b) all 4 cytokines (GM‐CSF + IFNγ+IL‐2 + TNFα+ among CD4+), (c) IFNγ and TNFα (GM‐CSF‐IFNγ+IL‐2‐TNFα+ among CD4+) and (d) IFN‐γ only (GM‐CSF‐IFNγ+IL‐2‐TNFα‐ among CD4+) after stimulation with WT and Omicron BA.2 whole spike proteins, and whole nucleocapsid. Sidak's multiple comparison correction was used for comparing young versus old, and Tukey's multiple comparison correction was used for comparing time‐points within the age groups. *p* values were determined by two‐way ANOVA with Sidak's and Tukey's multiple comparison corrections; *p < 0.05; **p < 0.01; ***p < 0.001; ****p < 0.0001. Bars represent mean ± standard deviation. (e) Correlation between percentage of spike‐specific CD4+ cells expressing IFNγ and neutralisation against WT and Omicron BA.2 antigens at day 100 in young (*n* = 15) and old (*n* = 14) vaccinees. Pearson's normally distributed correlation coefficient for linear data was used.

The changes in the proportion of polyfunctional CD4+ cells expressing multiple cytokines were assessed by Boolean analysis following vaccination in both groups. The proportion of polyfunctional CD4+ cells expressing at least 3 cytokines was increased in vaccinated young adults after stimulation with WT or Omicron spike (D0 vs. D100: WT spike 4.1% vs. 19.2%, *p* = 0.046; Omicron spike 4% vs. 20.2%, *p* = 0.010; NC 4.1% vs. 9.8%) whereas the frequency of these cells declined in the older adults (D0 vs. D100: WT spike 11.3% vs. 2.1%; Omicron spike 20% vs. 1.2%; NC 10% vs. 3.6%, Figure 2a). Expansion of two cytokines‐producing cells was observed following vaccination in both groups after WT and Omicron antigen stimulations.

A significant increase in the frequencies of GM‐CSF + IFNγ+IL2 + TNFα+ (WT, mean ± SD: D0 0 vs. D100 0.002 ± 0.002, *p* = 0.011; Omicron: D0 0 vs. D100 0.002 ± 0.002, *p* = 0.016, Figure 2b), IFNγ+TNFα+ (WT: D0 0.002 ± 0.003 vs. D100 0.019 ± 0.013, *p* = 0.010; Omicron: D0 0.002 ± 0.002 vs. D100 0.025 ± 0.016, *p* = 0.0034, Figure 2c) and IFNγ+ T‐cells (WT: D28 0.00027 ± 0.00050 vs. D100 0.002 ± 0.002, *p* = 0.025; Omicron: D0 0.004 ± 0.006 vs. D100 0.012 ± 0.006, *p* = 0.039, Figure 2d) was observed in the young adults at D100 compared to baseline after stimulation with WT or Omicron spike. However, the expansion of these T‐cell subsets was not apparent in the older adults with any of the antigen stimulations. The frequencies of these T‐cell subsets after Omicron stimulation were also significantly higher in the young adults than the old at D100 (GM‐CSF + IFNγ+IL2 + TNFα+, mean ± SD: young 0.002 ± 0.002 vs. old 0.0001 ± 0.0002, *p* = 0.027, Figure 2b; IFNγ+TNFα+: young 0.025 ± 0.016 vs. old 0.003 ± 0.006, *p* = 0.0034, Figure 2c; and IFNγ+: young 0.012 ± 0.006 vs. old 0.002 ± 0.002, *p* = 0.0015, Figure 2d). There were no significant changes in these T‐cell subsets from baseline to D28 with antigen stimulation.

In addition, we observed positive correlations between WT and Omicron spike‐stimulated IFNγ+ expressing CD4+ cells with neutralising antibody responses against the WT (*r* = 0.74, *p* = 0.01) and Omicron variants (*r* = 0.60, *p* = 0.049) respectively at D100 (Figure 2e). Conversely, this correlation was negative, though not significantly so, in the older adults for either variant.

In response to WT spike stimulation of the CD8+ population, young adults only showed expansion of TNFα+ and IL‐2+ − expressing CD8+ T‐cells, and at D28 was significantly higher than that observed in the older adults. A higher frequency of TNFα+ and IL‐2+ − expressing CD8+ T‐cells was also observed in the young adults compared to the older adults at D100 after Omicron spike stimulation but this was not significant (Figure S4).

In summary, we found that community‐dwelling older adults who completed the BNT162b2 primary vaccination regime exhibited weaker neutralisation responses to SARS‐CoV‐2. This finding may be accounted for by the lack of Tfh cell expansion which is central in helping B‐cells generate high‐affinity antibodies, long‐lived plasma cells, and memory B‐cells (Luo & Yin, 2021; Vella et al., 2017). Interestingly, we found that antigen‐specific IFNγ‐secreting CD4+ T‐cells to wild‐type and Omicron antigens were positively correlated with neutralising antibody responses. This relationship was markedly absent in older adults. This relative IFNγ‐secreting CD4+ T cell deficiency in older adults to the COVID‐19 mRNA vaccination response has not been shown previously and may explain the poorer vaccination response in older adults.

Neutralisation against the Omicron variants was not observed in both groups after vaccination. There are two possible reasons for this. First, the Omicron variants display mutations in the Spike N‐terminal and receptor‐binding domains that are associated with immune escape (Liu et al., 2022; Sokal et al., 2022). Second, our study examined the immune response after only two vaccination doses, and studies have shown that the protection against the Omicron variant may improve after the third dose of vaccine (Xia et al., 2022). Development of IgG+ MBCs after vaccination was diminished in older adults compared to young. Expansion of IgG+ MBCs and reduction of IgA+ MBCs have been previously reported in response to vaccination (Goel et al., 2021; Sureshchandra et al., 2021), with a decreased humoral response to vaccinations with age which could be due to a reduced size and function of the germinal center response (Lee & Linterman, 2022). Additionally, the increase in CD4+ T cells expressing CCR6 post‐vaccination was only seen in the young. CCR6 expression has been shown to be involved in the regulation of Th17 cell function and migration to the inflamed bile ducts in liver injury (Oo et al., 2012) and sites of inflammation in EAE mouse models (Yamazaki et al., 2008). Our findings are supported by previous literature in younger adults (median age 40 years old), which showed an expansion of CD4+ T cells skewed toward a Th17 phenotype in circulation (Sureshchandra et al., 2021) and in nasal swab samples (Ssemaganda et al., 2022) following two COVID‐19 mRNA vaccination doses. Interestingly, the lack of Th17 increase has not been reported in older adults for the COVID‐19 mRNA vaccination, and can potentially explain the reduced B cell response seen. In contrast to B and CD4+ T‐cells, CD8+ T‐cells showed no significant changes in the two groups after vaccination (without antigen stimulation) as participants at this point of the study did not have COVID‐19 infection. However, older adults were observed to exhibit more T_EMRA_ and less naïve CD8+ T‐cells. A higher proportion of CD8^+^ T_EMRA_ and reduction in naïve CD8 T‐cells have been shown to be associated with a reduction in Spike‐specific T‐cell responses after vaccination in older individuals (Palacios‐Pedrero et al., 2022). Older adults have been found to mount impaired antigen‐specific CD8+ T cell responses, possibly attributable to intrinsic qualitative cellular defects and reduced naïve pool secondary to thymic involution, which in turn was associated with poor primary immune responsiveness *in vivo (*Briceño et al., 2016 *)*.

SARS‐CoV‐2 Spike‐specific CD4+ T‐cells were shown to exhibit polyfunctionality, with younger adults showing greater expansion of CD4+ T‐cells expressing three or more cytokines after vaccination. In particular, polyfunctional CD4+ T‐cell subsets expressing IFNγ increased significantly in the vaccinated young adults in response to WT and Omicron spike stimulation but not in the older adults. The increase in polyfunctional T‐cells may indicate enhancements in recall functions and immune protection, and lower numbers in older adults may contribute to suboptimal protection after vaccination. IFNγ‐only‐expressing CD4+ T‐cells were positively correlated with neutralising response to WT and Omicron in young adults at D100, which has not been previously described. This relationship is markedly absent in older adults. This finding reiterates the role of IFNγ for COVID‐19 mRNA vaccine‐induced protection and relative IFNγ‐deficiency in older adults could explain the poorer vaccination responses observed. CD8+ T‐cells expressing IL‐2 and TNFα were the main Spike‐specific responses in young vaccinees but not in older adults. No differences were observed in IFNγ‐expressing and polyfunctional CD8+ cells after vaccination between both groups. CD8 responses to antigen stimulation are less pronounced because whole protein antigens, used in this study, undergo processing by host APCs via the exogenous pathway which favours MHC class II presentation and CD4+ T‐cell stimulation, whereas CD8^+^ T‐cell responses require antigen processing by the endogenous pathway, leading to MHC class I presentation. “Cross‐priming” of CD8+ T‐cell responses can occur in the presence of exogenous antigen, but this process is generally inefficient. Taken together, the findings also highlight the importance of T‐cell immunity in the absence of B‐cell responses or against divergent variants, such as Omicron, which escape neutralising antibodies.

The Th1 axis is implicated in viral defence and has been previously described in studies on immunosenescence (Crooke et al., 2019; Nikolich‐Žugich, 2018), with a shift from type 1 predominant to type 2 cytokine response with ageing (Sandmand et al., 2002). IFNγ is the pivotal cytokine in inducing Th1 responses and is key in the defence against intracellular pathogens such as mycobacteria and viruses. Through enhancement of MHC I and II expression on antigen‐presenting cells and macrophage phagocytic function, IFNγ upregulates anti‐viral clearance, inhibition of cellular proliferation and effects on apoptosis, activation of microbicidal effector functions, immunomodulation, and leukocyte trafficking (Schroder et al., 2004). However, previous studies have conflicting results of the relationship between IFNγ and age in animal (Haynes et al., 2003; Kim et al., 2013) and human studies (Ouyang et al., 2002; Zhou et al., 2014). Some have described aberrant upregulation of IFN‐I and ‐III leading to IFNγ activation in tissue ageing (Cao, 2022), while others show transcriptional deficiencies in IFNγ‐signalling (Herrero et al., 2001), impaired IFN‐I response and defective T‐cell activation to dampen IFNγ production (Feng et al., 2021). Hence, there remains much to be discovered regarding impactful impairments in IFNγ production or signalling during aging (Feng et al., 2021).

The relative IFNγ‐secreting CD4+ T cell deficiency we report in our study may be a potential mechanism for this immunological shift to type 2 responses. The consequent susceptibility to infections is clearly demonstrated in tuberculosis (TB) and varicella‐zoster virus (VZV) reactivation, two key conditions that arise in ageing, as well as influenza. First, the TB epidemic is most prevalent in older adults, with a peak in those 65 years and older. This rise in incidence is mostly linked to reactivation, which has been attributed to immunosenescence. TB mortality rate remains the highest in older patients, specifically in those 70 years and older (Caraux‐Paz et al., 2021). CD4+ T‐cells secreting IL‐2 and IFNγ is a potential correlate of protective immunity to TB (Lalvani & Millington, 2008), and humans deficient in the IFNγ gene or receptor show enhanced susceptibility to mycobacterial infections (Ottenhoff et al., 1998). These findings highlight the importance of IFNγ as a mediator of macrophage activation and its necessity in host immunity against TB. Second, VZV reactivation results from a decline in virus‐specific cell‐mediated immunity (Arvin, 1992), which is seen in immunosenescence. VZV reactivation correlates with a decline in IFNγ‐producing immune cells. From mouse models, IFNγ inhibits VZV replication in a cell line‐dependent manner via VZV gene expression inhibition after the immediate early stage of infection (Shakya et al., 2019). Therefore, lower levels of IFNγ with age result in immune escape and VZV reactivation. Third, influenza causes more severe respiratory disease in older adults. Efforts into producing a more effective influenza vaccine have found that an intranasal nanoparticle vaccination elicited a persistent, polyfunctional CD4+ T cell response producing IFNγ and TNFα in murine models, which mediates survival from a lethal challenge with H1N1 influenza virus (Nelson et al., 2021). This suggests an important protective role of IFNγ‐secreting CD4+ T cells in influenza which warrants further investigation in humans. Hence, these findings suggest that IFNγ is part of an immune risk phenotype which predicts remaining longevity in older adults (Ouyang et al., 2002) via modulating immunosenescence.

Our sample size is small and interrogation on a larger scale is needed to confirm our findings. This small sample size is due to multiple logistical constraints. The vaccination roll‐out was pushed rapidly, with priority given to those above 70 years old. Hence, a large proportion of older adults were vaccinated before ethical approval was able to be obtained. Additionally, many older adults declined participation in view of the multiple site visits required during the ongoing pandemic, and home visits were highly discouraged due to the infection risk. Recruitment was stopped when the government announced for vaccination‐differentiated measures in the community as it was deemed that those left unvaccinated were unlikely to accept vaccination. As our sample size was small, we could not control for potential confounders on the immune system such as muscle function. This exploration may warrant a larger study. In view of our sample quantity restrictions, we were unable to analyze SARS‐CoV2 spike‐ and nucleocapsid‐specific B cell activity, as done previously (Muecksch et al., 2022). Future studies with larger sample quantities would allow for analysis of antigen‐specific B cell activity in an age‐differentiated manner.

Altogether, we demonstrate that IFNγ is a key player in the age‐related attenuated T‐cell help and subsequent reduced B‐cell and neutralisation response to vaccination. This finding suggests IFNγ therapy may be promising in improving vaccination and infection outcomes in older adults. Further exploration into the underlying aetiology, such as compromised muscle function or obesity in older adults affecting IFNγ regulation, is needed. Hence, improving understanding of age‐differentiated vaccination response can lead to novel interventions to prevent or reverse its onset and increase healthspan in our older adults.

## AUTHORS CONTRIBUTIONS

VWTH conceptualised the study, obtained ethics approval, oversaw subject recruitment, analysed and interpreted the data and wrote the manuscript. LHB designed and performed the experiments, analysed and interpreted the data and wrote the manuscript. BS, RG, NYJT, XQ and KP conducted, analysed and interpreted the experiments. JZC and JC prepared the figures. ZJ oversaw subject recruitment. BK and VA conceptualised the study. SWF, LR and LFPN obtained ethics approval and oversaw subject recruitment. CWMO conceptualised the study, obtained ethics approval, oversaw subject recruitment and edited the manuscript. PAM conceptualised the study, interpreted the data and edited the manuscript. All authors have read and agreed to the published version of the final manuscript.

## FUNDING INFORMATION

This work was supported by the National University Singapore Infectious Diseases Translational Research Programme Seed Fund, the National University Hospital (NUH) COVID‐19 Adhoc Fund (ref: NUHSRO/2021/032/NUSMedCovid/08) and the Singapore National Medical Research Council COVID‐19 Research Fund (COVID19RF‐001; COVID19RF‐007; COVID19RF‐0008; COVID19RF‐060). Open access funding is provided by NUH. V.W. T. H is funded by the National Medical Research Council (NMRC) MOH‐RTF21jun‐0005. C.W.M.O. is funded by NMRC CSAINV21nov‐0003. L.R. and L.F.P. were supported by grants from the Singapore National Medical Research Council COVID‐19 Research Fund (COVID19RF‐0011; COVID19RF‐0018; COVID19RF‐0060), US Food and Drug Administration (#75F40120C00085), LR was supported by a Start‐up University Grant from the Singapore Ministry of Education (SUJ#022388‐00001).

## CONFLICT OF INTEREST STATEMENT

C.W.M.O. reports speaker fees from Qiagen travel support from MSD and grant from Institut Merieux outside this work.

## Supporting information


Appendix S1

## Data Availability

All data are included in the manuscript and Appendix S1. Demographic, clinical and immunological data are available from the authors upon reasonable request, due to privacy protection.

## APPENDIX 1

SCOPE Cohort Study Group: Siti Naqiah Amrun^8^, Yun‐Shan Goh^8^, Matthew Zi‐Rui Tay^8^, Angeline Rouers^8^, Zi Wei Chang^8^, Nicholas Kim‐Wah Yeo^8^, Yi‐Hao Chan^8^, Pei Xian Hor^8^, Chiew Yee Loh^8^, Yuling Yang^8^, Anthony Torres Ruesta^8^, Vanessa Neo^8^, Wendy Yehui Chen^8^, Estelle Yi‐Wei Goh^8^, Alice Soh‐Meoy Ong^8^, Adeline Chiew Yen Chua^8^, Samantha Nguee^8^, Yong Jie Tang^8^, Weiyi Tang^8^, Joel Xu En Wong^8^, Anna Loo, Liang Hui Loo, & Andrea Chua.
