# Supplementary material for: Relative deficiency in interferon‐γ‐secreting CD4+ T cells is strongly associated with poorer COVID‐19 vaccination responses in older adults
Source: Aging Cell. 2024 Feb 5;23(4):e14099. doi: 10.1111/acel.14099 (PMC11019126; doi:10.1111/acel.14099)
Supplement: Supplementary file 1 — Appendix S1 [file ACEL-23-e14099-s001.docx]

**Supporting Information**

**Experimental Procedures**

Study design and participants

This was a prospective longitudinal study on community-dwelling volunteers between the ages of 25 to 35 years (interquartile range, IQR), and 70 to 73 years (IQR) who have not received any COVID-19 vaccination. Recruitment was from March to December 2021. Participants all consented to be vaccinated with the BNT162b2 primary vaccine regime at a three-week dosing interval and were willing to comply with the study procedures. None of the enrolled participants had recent, current or persistent infectious diseases including COVID-19 infections determined by polymerase chain reaction (PCR), any known malignancies, or on treatment with any immunosuppressants. Presence of COVID-19 infection was also confirmed by nucleocapsid test of infection. Participants were followed up for 100 days after receiving their first dose of the BNT162b2 vaccine. Participants provided blood samples prior to vaccination (day 0), before the second dose vaccination (day 28) and two months after the second vaccination (day 100).

A subset of participants underwent functional assessment (eg Barthel Index for basic activities of daily living^35^, Lawton’s instrumental activities of daily living (IADL) scale^36^, maximum handgrip strength (HGS), gait speed, short physical performance battery (SPPB)), cognitive tests (Montreal cognitive assessment (MoCA)^37^) and mood using patient health questionnaire-9 (PHQ-9)^38^, nutrition using body mass index (BMI) and Mini nutritional assessment short form (MNA-SF)^39^, health-related quality of life using Euro-QoL EQ-5D-5L questionnaire^40^, and FRAIL score measuring fatigue, resistance, aerobic, number of illnesses, and loss of weight with a maximum score of 5 was used to assess frailty^41^. Medical questionnaire was conducted for baseline demographics, medical history and medications. Blood samples were collected for all participants at D0, D28 and D100.

Quantitative Enzyme-Linked Immunosorbent Assay (ELISA)

The concentrations of anti-Spike immunoglobin G (IgG), anti-Spike immunoglobulin A (IgA) and anti-Nucleocapsid (NC) IgG antibodies in plasma samples were estimated using quantitative ELISA^42^ as described previously. To estimate the titres of anti-spike and anti-nucleocapsid IgG, an receptor-binding domain (RBD)-specific human monoclonal IgG antibody (LSI-COVA-015, isolated from COVID-19 convalescent patient) and a NC-specific monoclonal IgG antibody (LSI-COVANC-D generated from hybridoma cloning^43 44^) were diluted to a series of concentrations ranging from 1 ng/mL to 1 µg/mL and added at 100 µL/well. Similarly, an anti-Spike IgA antibody (PD6 generated via Phage-Fab display technology) was serially diluted to estimate the anti-Spike IgA titres in the plasma samples. Standard curves were constructed using the reference antibodies from 100 ng/mL to 1 ng/mL, and the concentration of antigen-specific IgG and IgA antibodies in plasma samples were calculated via interpolation.

Angiotensin-converting Enzyme 2 (ACE2)-RBD binding inhibition ELISA

Neutralizing antibody responses were tested using an ACE-2 inhibition assay to SARS-CoV-2 variants; Wuhan-Hu-1, Alpha, Beta, Gamma, Delta and Omicron B.1.1.529 and BA.2. A cut-off of 40% inhibition and below was considered inadequate neutralization. This ACE2 inhibition assay was used as it significantly reduced the time taken to complete while achieving a similar sensitivity and specificity to the pseudovirus neutralisation test (PVNT) (**sFig2, sTable 2**). The ACE2 inhibition assay also allowed for analysis of neutralising capacity against multiple RBD variants in one assay compared to PVNT which only allows for one variant per test. Wuhan-Hu-1, Alpha, Beta, Gamma, and Delta RBD were diluted to 1 µg/mL and coated at 50 µL/well onto 96-well flat-bottom maxi-binding immunoplates (SPL Life Sciences, Gyeonggi-do, South Korea, #32296) with incubation at 4 °C overnight. Wuhan-Hu-1 RBD and Omicron RBD were also coated at 2 µg/mL for calibration of Omicron RBD results to account for the difference in coating concentrations. The plate was washed three times with washing buffer (1x phosphate buffered saline (PBS) with 0.05% Tween-20) and blocked with blocking buffer (3% bovine serum albumin in washing buffer) at 350 µL/well for 60-minutes. After washing the plate, plasma samples were diluted 5-times in blocking buffer, added at 50uL/well and incubated for 1 hour. A negative control (5-times diluted heat-inactivated fetal bovine serum (FBS)) and a positive control (ACE2-fragment crystallizable (Fc) at 100 µg/mL in negative control) were included for each variant in each plate. The plate was then washed and incubated with the secondary antibody, ACE2-Peroxidase (conjugated using Peroxidase-labelling kit-NH_2_, Abnova, Taipei, Taiwan, #KA0014), at 50 µL/well for 50 minutes, protected from light. A concentration of 600 ng/mL and 300 ng/mL of the ACE2-Peroxidase was used to detect for Omicron and the other RBD variants respectively. After another plate wash, 3,3′,5,5′-Tetramethylbenzidine (TMB) substrate (Thermo Fisher Scientific, Waltham, MA, United States of America (USA), #34029) was added at 100 µL/well and incubated for 3 minutes. The reaction was stopped with 50 µL/well 1 M H_2_SO_4_ and optical density at 450 nm (OD_450_) was measured. Inhibition% was calculated using the following formula:

Inhibition% = (Readout (negative control)-Readout (sample))/(Readout (negative control) )×100%

Peripheral Blood Mononuclear Cells (PBMCs) Preparation

PBMCs were isolated by density-gradient sedimentation using Ficoll-Paque Plus (Cytiva, Washington DC, USA). Blood samples were collected in Vacutainer blood collection tubes with EDTA (BD Biosciences) and centrifuged for 30 min at 400g to separate the cellular fraction and plasma. The plasma was removed from the PBMC fraction and stored at −80°C. Isolated PBMCs were cryopreserved in cell recovery media containing 10% dimethyl sulfoxide (DMSO) (Gibco), supplemented with 90% heat-inactivated FBS (Hyclone Laboratories, Logan Utah, USA) and stored in liquid nitrogen until use for the assays. Cryopreserved PBMCs were thawed in pre-warmed RPM1 1640 (Gibco, Thermo Fisher Scientific, Waltham, MA, USA). After centrifugation, cells were washed twice before use in subsequent assays.

Flow Cytometry Analyses

High dimensional flow cytometry for B and T-cell subsets was conducted to measure magnitude and kinetics of response to the COVID-19 vaccination over time. Frozen PBMCs were thawed, washed in fluorescence-activated cell sorting (FACS) buffer (2% FBS, 1 mM ethylenediaminetetraacetic acid (EDTA) in PBS), and counted before surface staining using the following panels (**sTables 3**), including Live/Dead Fixable Near IR fluorescent dye (Gibco, Thermo Fisher Scientific, Waltham, MA, USA) to exclude non-viable cells. Samples were then resuspended in 300uL FACS buffer and acquired on the Cytek Aurora flow cytometer. Data were analysed using FlowJo v10 (TreeStar, Ashland, OR, USA). The detailed gating strategies are described in **sFig 1**.

Expression and purification of SARS-CoV-2 antigens and receptors

SARS-CoV-2 Spike proteins were purified as described elsewhere^45^. Primers used are listed in **sTable 4**. The gene encoding SARS-CoV-2 NC (Bio Basic Asia Pacific, Singapore) was cloned into pNIC28 and expressed in BL21 (DE3). Bacterial cells were cultured at 37°C until the optical density reached 0.6, and induced by adding Isopropyl ß-D-1-thiogalactopyranoside (IPTG) at a final concentration of 0.5 mM. After overnight incubation at 16 °C, cells were harvested by centrifugation. Cell pellets were resuspended in 50 mM tris(hydroxymethyl)aminomethane (Tris), 500 mM NaCl, pH 8.0 (buffer A) and lysed by sonication. After centrifugation, the supernatant containing the NC protein was harvested and incubated with cOmplete^TM^ his tag purification resin (Roche, Basel, Switzerland) at 4 °C for 1 hour. The resin was then washed with buffer A containing 25 mM imidazole and resin-bound NC was eluted with 50 mM Tris, 500 mM NaCl and 500 mM imidazole, pH8.0. Purified NC was concentrated using vivaspin concentrators with 30 kDa cut-off (GE healthcare, Chicago, USA) and buffer exchanged with 20 mM Tris, 300 mM NaCl, pH 8.0 using zeba^TM^ spin desalting column with 7k molecular weight cut-off (MWCO) (Thermo Fisher Scientific, Waltham, MA, USA).

Antigen-specific T-cell response to vaccination

PBMCs are stimulated with purified SARS-CoV-2 spike wild-type and Omicron BA.2, as well as NC proteins at a final concentration of 5ug/mL 200uL of cell suspension (1.5 × 10^6^ PBMCs per well) was seeded in 96-well flat-bottom plates (Corning, New York, USA) and incubated with the purified proteins in the presence of co-stimulatory antibodies, anti-CD28 and -CD49d at a final concentration of 1ug/mL (eBioscience, Thermo Fisher Scientific, Waltham, MA, USA). Co-stimulatory antibodies without proteins were added to control wells. After 3 hours of incubation, brefeldin A was added at a final concentration of 1ug/mL to each well and incubated for another 16 hours.

After stimulation, the cells were washed and stained with Live/Dead Fixable Near-IR fluorescent dye. The cells were then stained with surface markers as listed in **sTable 3**. After this, the stained cells were fixed with IC fixation buffer (Thermo Fisher Scientific, Waltham, MA, USA) for 30 min at 4 °C, washed twice with permeabilization buffer (Thermo Fisher Scientific, Waltham, MA, USA), and stained with intracellular markers. Samples were then resuspended in 300uL FACS buffer and analysed on the Cytek Aurora flow cytometer. Antigen-specific responses were quantified as the frequency of cytokine-expressing cells in stimulated samples, with background subtraction from paired controls with no antigen stimulation. Proportions of multiple cytokine-expressing CD4+ T-cells (granulocyte-macrophage colony-stimulating factor GM-CSF, interferon-gamma IFNγ, interleukin-2 IL-2 and tumour necrosis factor-alpha TNF-α) were assessed by Boolean analysis. The detailed gating strategies are described in **sFig 1**. The total antigen responsive cells were defined as CD4+CD154+ T-cells. The frequency of expression of the different cytokines on the antigen specific CD4 T-cells was expressed as a as a proportion of the total antigen specific CD4 T-cells.

Statistical analyses

Descriptive analyses of demographic and clinical data are presented as mean and standard deviation when continuous and as frequency and proportion (%) when categorical. Differences between continuous and categorical data were tested using Student T-test and Chi-square tests, respectively. Two-way ANOVA and linear regression were used to model the association between age groups and neutralization by vaccine-elicited antibodies, B and T-cell subsets and antigen-specific T-cell responses at different timepoints. The Pearson’s normally distributed correlation coefficient for linear data were reported. Statistical analyses and graphs were done using Stata version 14 (Stata Corp LP, College Station, TX, USA) and GraphPad Prism software version 9 (GraphPad Software, La Jolla, CA, USA). Significance was determined to be p<0.05. Results were reported to 2 decimal places.

**sTable 1. Baseline demographics and characteristics of the cohort by age.**

|  | **Young** (n=15) | **Old** (n=14) | **P value*** |
| --- | --- | --- | --- |
| **Age**, median (IQR) | 31 (25-35) | 72 (70-73) | <0.01 |
| **Male**, n (%) | 8 (53.3) | 7 (50) | 0.72 |
| **Chinese ethnicity**, n (%) | 14 (93.3) | 14 (100) | 0.17 |
| **Medical conditions**^a^, n (%) | 0 (0) | 4 (57.1) | <0.01 |
| **BMI**^a^ (kg/m2), median (IQR) | 22.4 (19.3-26.4) | 22.2 (22.0-24.2) | 0.88 |
| **MNA-SF**^a^, median (IQR) | 14 (14-14) | 14 (13-14) | 0.41 |
| **Barthel score**^a^, median (IQR) | 100 (100) | 100 (100) | 0.14 |
| **Lawton scale**^a^, median (IQR) | 8 (8-8) | 8 (7-8) | 0.04 |
| **Max HGS**^a^ (kg), median (IQR) | 29.8 (22.6-37.2) | 26.7 (23.2-35.2) | 0.81 |
| **TUG**^a^ (s), median (IQR) | 10.1 (9.9-11.1) | 17.4 (13.7-18) | <0.01 |
| **STS**^a^ (s) | 8.22 (8-10.22) | 17.5 (13.21-21.1) | <0.01 |
| **SPPB**^a^ score | 11 (10-12) | 7 (7-8) | <0.01 |
| **FRAIL**^a^ score, median (IQR) | 0 (0) | 0 (0-1) | 0.03 |
| **MoCA**^a^, median (IQR) | 28 (25-30) | 26 (20-28) | 0.13 |

BMI, body mass index; FRAIL, screening test for frailty; HGS, Handgrip Strength; IQR, interquartile range; MNA-SF, M.I.N.I. Nutritional Assessment-Short Form; MoCA, Montreal Cognitive Assessment; SPPB, short physical performance battery; STS, 5-times Sit-to-Stand; TUG: Timed-Up-and-Go.

^a^ Values calculated from 15 young adults and 7 older adults.

*p-values were determined by paired T-test and Chi-square test for continuous and categorial data respectively.

**sTable 2. Performance of neutralizing response by ACE-2 inhibition assays**

|  | **Sensitivity%** | **95%CI** | **Specificity%** | **95%CI** | **Likelihood ratio** |
| --- | --- | --- | --- | --- | --- |
| >36.92 | 100.0 | 67.56% to 100% | 71.43 | 35.89% to 94.92% | 3.500 |
| >53.87 | 87.50 | 52.91% to 99.36% | 85.71 | 48.69% to 99.27% | 6.125 |
| >62.20 | 87.50 | 52.91% to 99.36% | 100.0 | 64.57% to 100.0% | - |
| >76.16 | 75.00 | 40.93% to 95.56% | 100.0 | 64.57% to 100.0% | - |

Sensitivity, specificity, and likelihood ratio of using ACE2 inhibition% as a predictor for an IC50 value of 150 IU/mL was evaluated. Figures close to the arbitrary threshold of 65% inhibition are reported.

**sTable 3. Cell panels for surface staining**

| **Marker** | **Fluorochrome** | **Clone** | **Manufacturer** | **Catalogue number** |
| --- | --- | --- | --- | --- |
| *B-cell and monocyte panel* | | | | |
| CD3 | APC-Cy7 | OKT3 | Biolegend, San Diego, California, USA | 317342 |
| CD14 | BV786 | Mphip-9 | BD, Sparks, MD, USA | 741034 |
| CD16 | efluor450 | eBioCB16 (CB16) | ebioscience, Thermo Fisher Scientific, Waltham, MA, | 48-0168-42 |
| CD19 | BV510 | SJ25C1 | Biolegend, San Diego, California, USA | 363020 |
| CD10 | PE Dazzle | HI10a | Biolegend, San Diego, California, USA | 312228 |
| CD21 | APC | Bu32 | Biolegend, San Diego, California, USA | 354906 |
| CD27 | BV650 | 323 | Biolegend, San Diego, California, USA | 302828 |
| CD38 | PE-Cy7 | HB-7 | Biolegend, San Diego, California, USA | 356608 |
| HLA-DR | FITC | L243 | Biolegend, San Diego, California, USA | 307604 |
| PD-1 | BV711 | EH12.2H7 | Biolegend, San Diego, California, USA | 329928 |
| IgD | BUV737 | 1A6-2 | BD, Sparks, MD, USA | 612798 |
| IgM | BUV395 | G20-127 | BD, Sparks, MD, USA | 563903 |
| IgG | Per-CP-Cy5.5 | G18-145 | BD, Sparks, MD, USA | 624353 |
| IgA | PE | IS11-8E10 | Miltenyi Biotech, Bergisch Gladbach, Germany | 130-113-476 |
| *T-cell and NK panel* | | | | |
| CD14 | APC-Cy7 | Mfp9 | BD, Sparks, MD, USA | 557831 |
| CD19 | APC-Cy7 | SJ25C1 | BD, Sparks, MD, USA | 557791 |
| CD3 | BUV395 | UCHT1 | BD, Sparks, MD, USA | 563546 |
| CD4 | BUV496 | SK3 | BD, Sparks, MD, USA | 612936 |
| CD8 | PerCP-Cy5.5 | SK1 | BD, Sparks, MD, USA | 565310 |
| CD25 | BV421 | M-A251 | BD, Sparks, MD, USA | 562442 |
| CD127 | FITC | HIL-7R-M21 | BD, Sparks, MD, USA | 560549 |
| CXCR5 | PE-Cy7 | RF8B2 | BD, Sparks, MD, USA | 624052 |
| ICOS | BUV737 | DX29 | BD, Sparks, MD, USA | 564778/749665 |
| CXCR3 (CD183) | PE | 1C6 | BD, Sparks, MD, USA | 557185 |
| CCR4 (CD194) | BV650 | 1G1 | BD, Sparks, MD, USA | 744140 |
| CCR6 (CD196) | APC | 11A9 | BD, Sparks, MD, USA | 560619 |
| PD-1 | BV711 | EH12.2H7 | Biolegend, San Diego, California, USA | 329928 |
| CD56 | BV786 | NCAM16.2 | BD, Sparks, MD, USA | 564058 |
| CD16 | efluor450 |  | eBioscience, Thermo Fisher Scientific, Waltham, MA, USA | 48-0168-42 |
| CD45RA | BV510 | HI100 | Biolegend, San Diego, California, USA | 304142 |
| CD57 | BV605 | QA17A04 | Biolegend, San Diego, California, USA | 393304 |
| KLRG1 | PE/Dazzle™ 594 | 14C2A07 | Biolegend, San Diego, California, USA | 368608 |
| CD27 | AlexaFluor700 | O323 | Biolegend, San Diego, California, USA | 302814 |
| TIM-3 | BUV615 | 7D3 | BD, Sparks, MD, USA | 752363 |
| CD28 | BUV805 | CD28.2 | BD, Sparks, MD, USA | 742037 |
| *Antigen-specific T-cell panel* | | | | |
| CD14 | efluor780 | 61D3 | eBioscience, Thermo Fisher Scientific, Waltham, MA, USA | 47-0149-42 |
| CD16 | efluor780 | eBioCB16 (CB16) | eBioscience, Thermo Fisher Scientific, Waltham, MA, USA | 47-0168-42 |
| CD19 | efluor780 | SJ25C1 | eBioscience, Thermo Fisher Scientific, Waltham, MA, USA | 47-0198-42 |
| CD27 | AlexaFluor700 | M-T271 | Biolegend, San Diego, California, USA | 356416 |
| IL-2 | APC | MQ1-17H12 | Biolegend, San Diego, California, USA | 500310 |
| CD154 | PE-Cy7 | 24-31 | Biolegend, San Diego, California, USA | 310832 |
| GM-CSF | PE | BVD2-21C11 | Biolegend, San Diego, California, USA | 502306 |
| CD3 | V500 | SP34-2 | BD Biosciences, Sparks, MD, USA | 560770 |
| CD4 | BUV496 | SK3 | BD Biosciences, Sparks, MD, USA | 612936 |
| CD8 | BUV805 | SK1 | BD Biosciences, Sparks, MD, USA | 612889 |
| IFN ɣ | BV421 | B27 | BD Biosciences, Sparks, MD, USA | 562988 |
| TNFɑ | BUV395 | MAb11 | BD Biosciences, Sparks, MD, USA | 563996 |
| HLA-DR | BV650 | G46-6 | BD Biosciences, Sparks, MD, USA | 564231 |
| CD38 | ECD (IOTest) | LS198-4-3 | Beckman-Coulter, California, USA | A99022 |

**sTable 4. Primers used to generate constructs for receptor-binding domain (RBD) variants**

| **Primer** | **Sequence** |
| --- | --- |
| K417N F | atattgctgattataattataaattaccagatga |
| K417 R | ttccagtttgccctggagcga |
| E484K F | aaaggttttaattgttactttccttta |
| E484K R | aacaccattacaaggtgtgct |
| A570D F | atgacactactgatgctgtccgt |
| A570D R | caatgtctctgccaaattgttgga |
| N501Y F | Ggtgttggttaccaaccatacaga |
| N501Y R | gtaagtgggttggaaaccatatgattg |
| L452R F | aggtatagattgtttaggaagtctaatctc |
| L452R R | gtaattataattaccaccaaccttagaa |
| K417TF | ctattgctgattataattataaattaccagatga |
| E484K 417R | taaaggaaagtaacaattaaaacctttaacaccattaca |
| N501Y 417F | caatcatatggtttccaacccacttacggtgttggttac |
| E484Q F | caaggttttaattgttactttccttta |
| T478K R | aacaccattacaaggtttgct |
| E484 F | gaaggttttaattgttactttccttta |
| L452Q F1 | ggtggtaattataattaccagtatagattg |
| L452Q R1 | aaccttagaatcaagattgttagaattc |

**sFigure 1. Gating strategy for flow cytometry analysis.**

**a b**

**
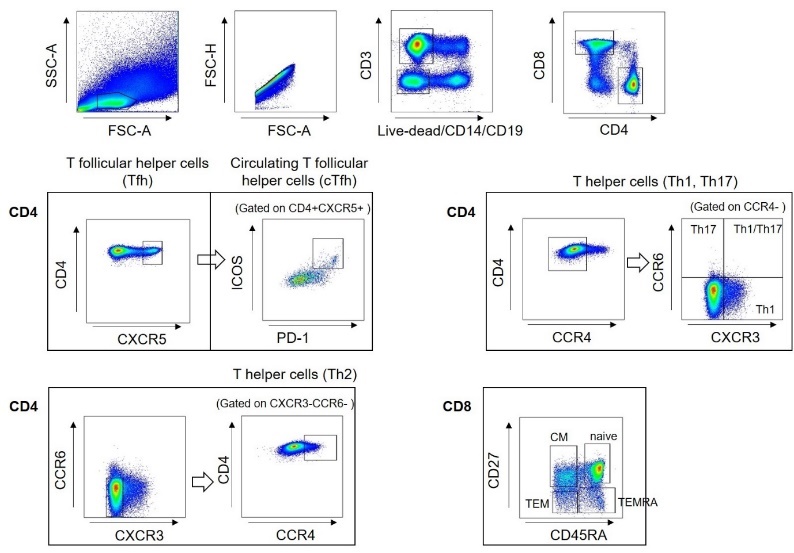
**
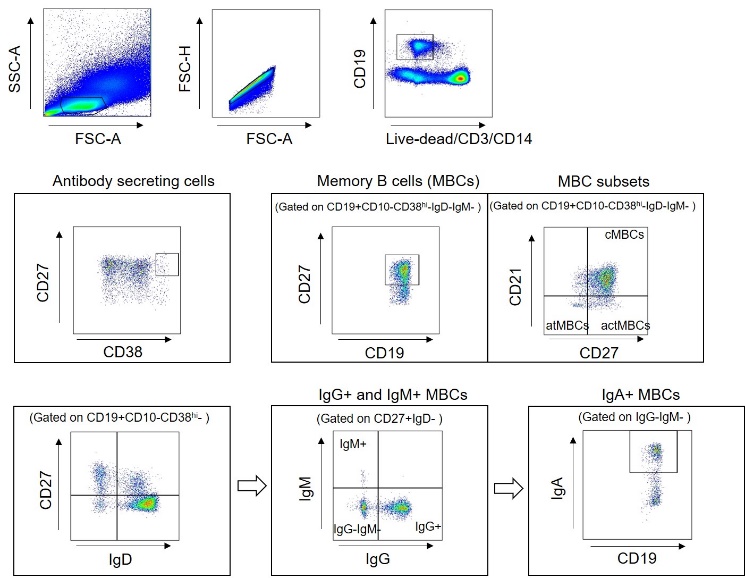


**c**

**
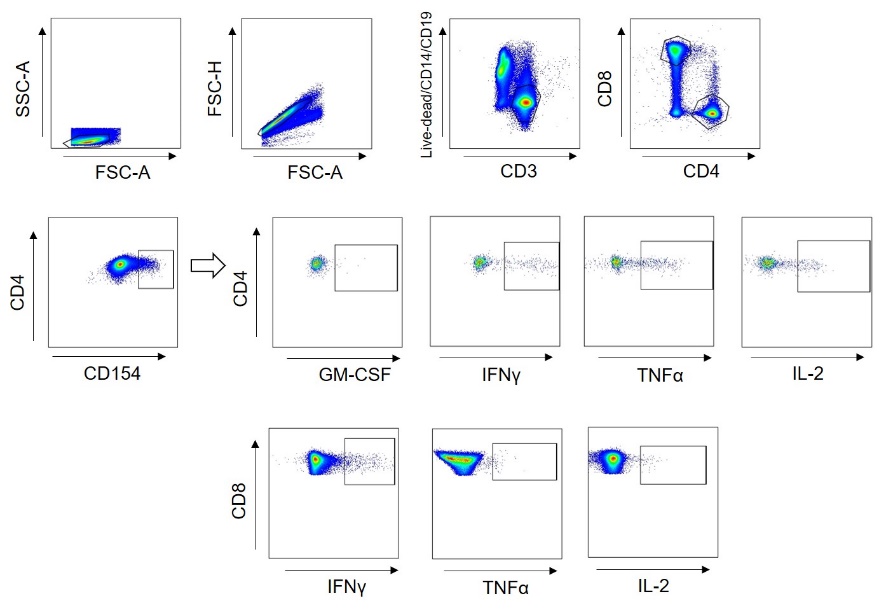
**

**a** Gating strategy to identify B-cell phenotypes. **b** Gating strategy to identify CD4 + and CD8 + T-cell phenotype and cytokine production. **c** Representative flow cytometry plots identifying CD4 + (middle panel) and CD8 + (bottom panel) producing GM-CSF, IFNγ, IL-2 and TNF-α in response to whole SARS-CoV2 antigens.

**sFigure 2. Comparison of neutralizing response by ACE-2 inhibition assays compared to PVNT**

**a**


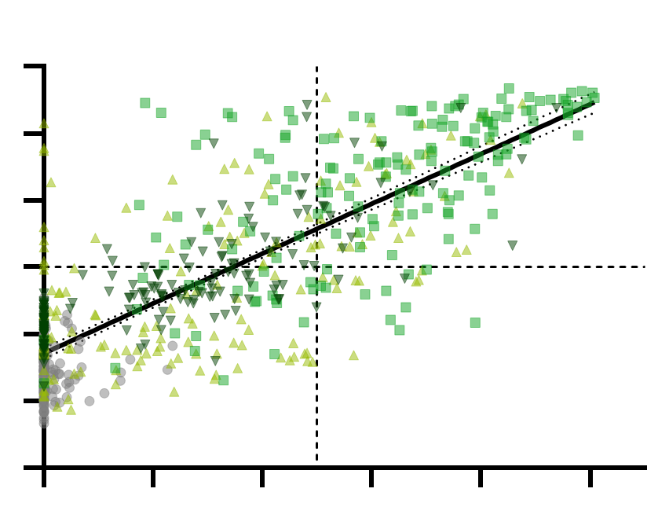


Pre-vaccine

Post-dose 1

Peak response

6 months post-vaccine

r = 0.8149

p < 0.0001

100

80

60

40

20

0

-20

**Inhibition% by ELISA**

0 20 40 60 80 100

**Neutralization% by PVNT**

**b**

100

80

60

40

20

0

**Sensitivity**

0 20 40 60 80 100

**100% Specificity%**


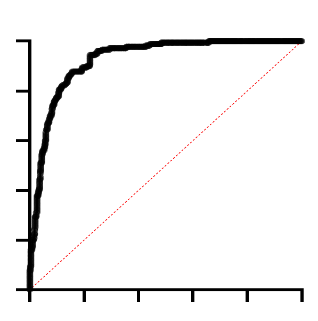


**a** Correlation between neutralizing response by PVNT and ACE2-RBD binding inhibition for Wuhan-Hu-1 RBD at four timepoints were modelled using simple linear regression. n = 672. Pearson’s correlation coefficients and p-values are shown*.* **b** Correlation between IC50, ACE2-RBD binding inhibition response, and PVNT. Using a threshold of 50% neutralization by PVNT, the predictability of ACE2-RBD binding inhibition response for defining neutralizers was evaluated with an ROC curve. n = 168.

**sFigure 3. Humoral responses to SARS-CoV2** **messenger ribonucleic acid (mRNA) vaccination.**


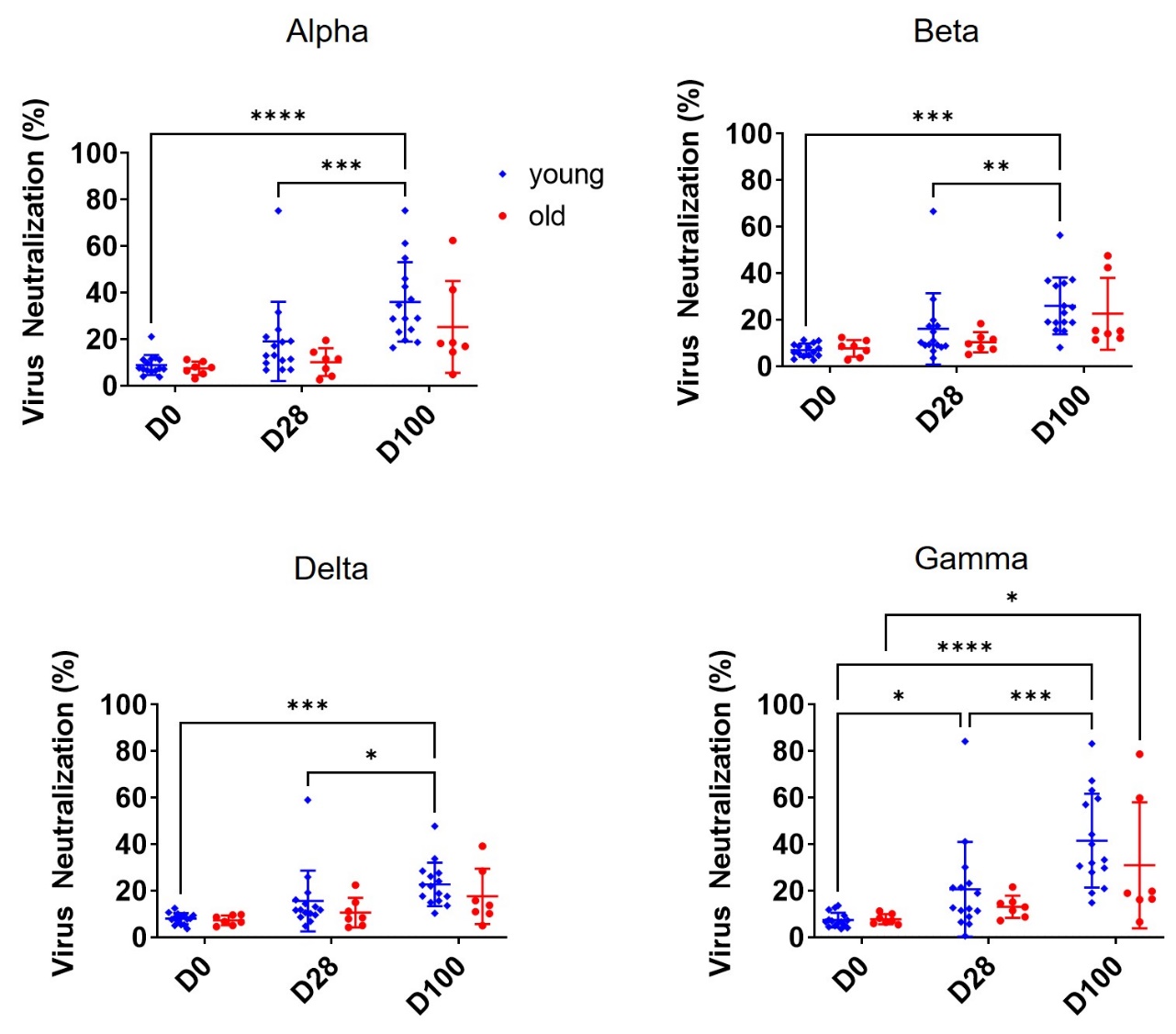


Virus neutralization assays against SARS-CoV2 Alpha, Beta, Delta and Gamma variants in young (n = 15) and old (n = 7) vaccinees. Sidak's multiple comparison correction was used for comparing young vs old, Tukey's multiple comparison correction was used for comparing time-points within the age groups. P values were determined by two-way ANOVA with Sidak’s and Tukey's multiple comparison corrections; *P < 0.05; **P < 0.01; ***P < 0.001; ****P < 0.0001.

**sFigure 4. Spike-specific CD8+ T-cell effector responses to SARS-COV2 mRNA vaccination.**

**a**

**
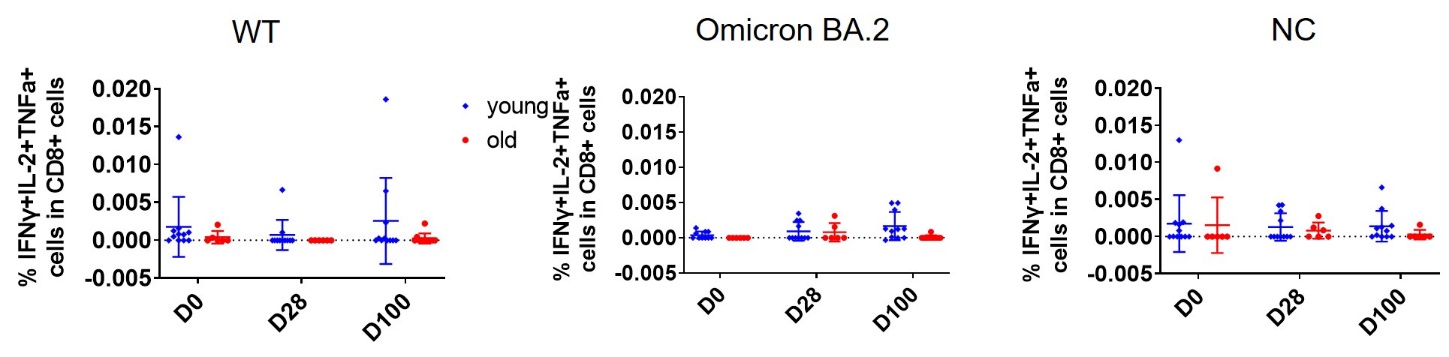
**

**b**

**
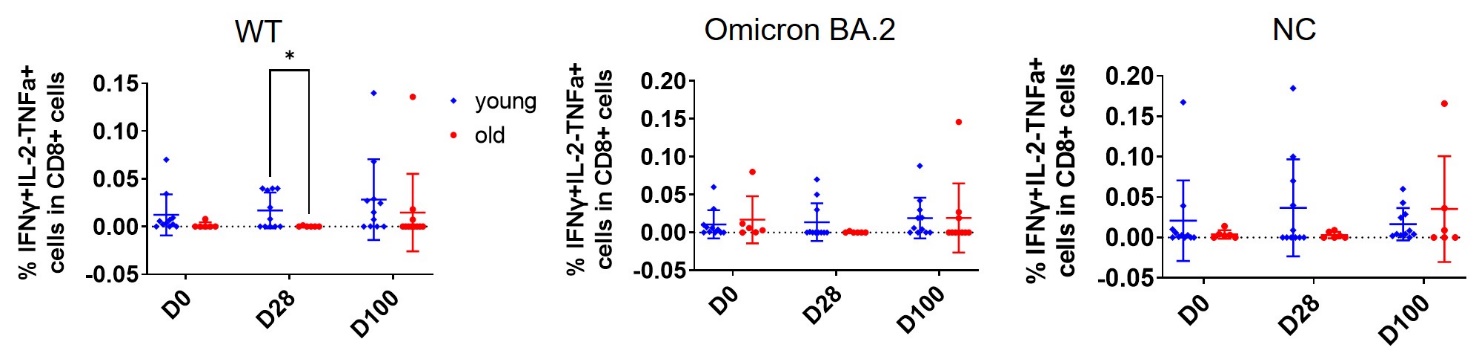
c**

**
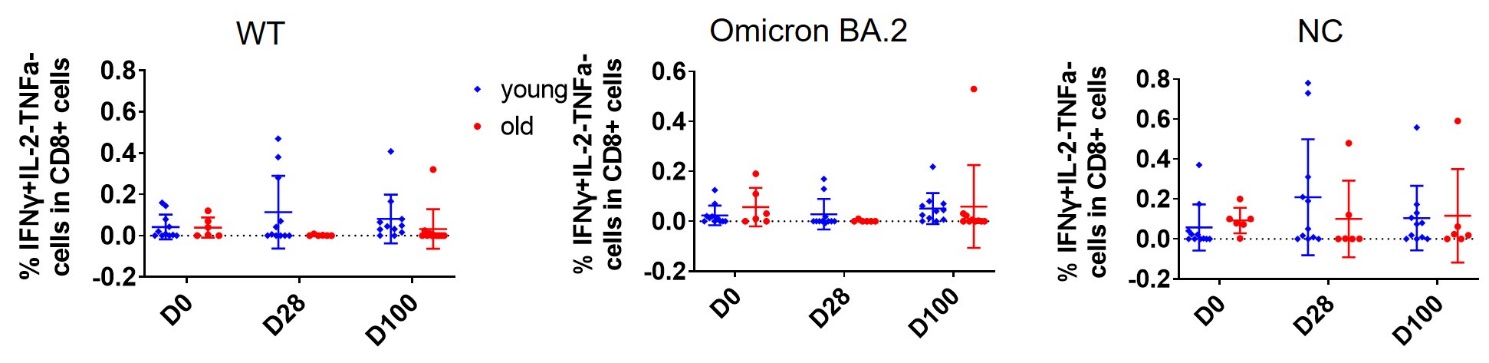
**

Frequencies of CD8+ T-cells expressing **a** 3 cytokines (IFNγ+IL-2+TNFα+ among CD8+) **b** IFNγ and TNFα (IFNγ+IL-2-TNFα+ among CD8+) and **c** IFN-γ only (IFNγ+IL-2-TNFα- among CD8+) after stimulation with WT and Omicron BA.2 whole spike proteins, and whole nucleocapsid in young (n = 15) and old (n = 14) vaccinees. Sidak's multiple comparison correction was used for comparing young vs old, Tukey's multiple comparison correction was used for comparing time-points within the age groups. P values were determined by two-way ANOVA with Sidak’s and Tukey's multiple comparison corrections.
